# Revealing molecular and cellular heterogeneity in hypopharyngeal carcinogenesis through single-cell RNA and TCR/BCR sequencing

**DOI:** 10.3389/fimmu.2024.1310376

**Published:** 2024-04-24

**Authors:** Cheng-Wei Tie, Ji-Qing Zhu, Zhan Yu, Li-Zhou Dou, Mei-Ling Wang, Gui-Qi Wang, Xiao-Guang Ni

**Affiliations:** ^1^ Department of Endoscopy, National Cancer Center/National Clinical Research Center for Cancer/Cancer Hospital, Chinese Academy of Medical Sciences and Peking Union Medical College, Beijing, China; ^2^ Department of Otolaryngology Head and Neck Surgery, Beijing Anzhen Hospital, Capital Medical University, Beijing, China; ^3^ Department of Endoscopy, National Cancer Center/National Clinical Research Center for Cancer/Cancer Hospital & Shenzhen Hospital, Chinese Academy of Medical Sciences and Peking Union Medical College, Shenzhen, China

**Keywords:** hypopharyngeal squamous cell carcinoma, carcinogenesis, dynamic transcriptomic mapping, tissue microenvironment, single-cell sequencing

## Abstract

**Introduction:**

Hypopharyngeal squamous cell carcinoma (HSCC) is one of the malignant tumors with the worst prognosis in head and neck cancers. The transformation from normal tissue through low-grade and high-grade intraepithelial neoplasia to cancerous tissue in HSCC is typically viewed as a progressive pathological sequence typical of tumorigenesis. Nonetheless, the alterations in diverse cell clusters within the tissue microenvironment (TME) throughout tumorigenesis and their impact on the development of HSCC are yet to be fully understood.

**Methods:**

We employed single-cell RNA sequencing and TCR/BCR sequencing to sequence 60,854 cells from nine tissue samples representing different stages during the progression of HSCC. This allowed us to construct dynamic transcriptomic maps of cells in diverse TME across various disease stages, and experimentally validated the key molecules within it.

**Results:**

We delineated the heterogeneity among tumor cells, immune cells (including T cells, B cells, and myeloid cells), and stromal cells (such as fibroblasts and endothelial cells) during the tumorigenesis of HSCC. We uncovered the alterations in function and state of distinct cell clusters at different stages of tumor development and identified specific clusters closely associated with the tumorigenesis of HSCC. Consequently, we discovered molecules like MAGEA3 and MMP3, pivotal for the diagnosis and treatment of HSCC.

**Discussion:**

Our research sheds light on the dynamic alterations within the TME during the tumorigenesis of HSCC, which will help to understand its mechanism of canceration, identify early diagnostic markers, and discover new therapeutic targets.

## 1 Introduction

The hypopharynx is crucial for physiological functions like swallowing and speech. Hypopharyngeal squamous cell carcinoma (HSCC) often develops unnoticed, leading to late-stage diagnoses. Despite advanced treatments, the 5-year survival rate is below 40%, marking it as one of the most severe malignancies in the head and neck region (1). Squamous cell carcinoma is the predominant form of hypopharynx, strongly linked to smoking and alcohol (2). The transformation from normal mucosa to cancer, involving stages of low-grade intraepithelial neoplasia (LGIN) and high-grade intraepithelial neoplasia (HGIN), is poorly understood due to the complexity of signaling networks and molecules involved (3). Thus, studying the cellular and molecular alterations in HSCC development is vital for understanding its molecular mechanisms, identifying diagnostic markers, and discovering therapeutic targets.

Understanding the biological mechanisms of HSCC carcinogenesis requires detailed characterization of the molecular, cellular, and acellular components involved in cancerous transformation, which exhibit pronounced spatial and temporal heterogeneity throughout tumor initiation and progression (4). This heterogeneity is marked by the emergence of distinct cellular entities, each with unique molecular signatures and functional differences (5).

Traditional transcriptome sequencing methods, which average RNA expression, can mask the disparities in gene expression among diverse cells within a group. The advent of single-cell RNA sequencing (scRNA-seq) has overcome this, allowing for the extraction and preparation of RNA libraries at the single-cell level and providing comprehensive transcriptomic insights. This technology aids in identifying cell types, states, and functions, and illuminates cellular heterogeneity and evolutionary pathways. Integrated with immunome library analysis, it offers a complete examination of the T-cell receptors (TCR) and B-cell receptors (BCR) of all immune cells, providing a detailed immune profile of gene expression in specific tissues or diseases. This analysis is crucial for uncovering tumor heterogeneity, tracing cell lineage, and understanding the complex mechanisms of tumor clonal evolution, paving the way for early detection, therapeutic stratification, prognostic evaluation, and monitoring of recurrence (6).

In this study, we explored the multi-stage carcinogenesis of hypopharyngeal mucosa by conducting scRNA-seq on tissue specimens from various pathological stages, aiming to analyze the composition and expression variations of cells within different tissue microenvironments (TME). This approach, simulating the progression of hypopharyngeal carcinogenesis, aspires to construct a comprehensive dynamic transcriptome map to depict the evolution of cellular and molecular expressions throughout tumorigenesis. The insights gained are expected to elucidate the trajectory and potential molecular mechanisms of hypopharyngeal carcinogenesis and reveal key regulatory molecules, aiding in the identification of predictive molecular markers for hypopharyngeal carcinogenesis, thereby contributing significantly to the advancement of HSCC analysis.

## 2 Materials and methods

### 2.1 Patient recruitment and sample collection

From January to February 2023, five male patients, median age 57 (55-63 years), with HSCC were recruited from the Cancer Hospital of the Chinese Academy of Medical Sciences. The study received approval from the hospital ethics committee (number: 22/454-3656), with informed consent obtained from each patient before examination. None had received any treatment (radiation, chemotherapy, or surgery) or had a history of other tumor diseases. Before treatment, all underwent laryngoscopy and gastroscopy. Based on laryngoscopic findings, multiple biopsies were taken from different hypopharyngeal regions of the patients, yielding nine samples: four from the left pyriform sinus, three from the right, and two from the posterior hypopharyngeal wall. Each sample was bifurcated; a fragment was preserved in 10% formalin for routine pathology, and the remainder was used for scRNA-seq and TCR/BCR-seq. Two experienced, blinded pathologists conducted the pathological assessments, agreeing on the final diagnoses. Histopathological diagnosis is the diagnostic gold standard. The final pathological diagnoses were two cases of normal squamous epithelial tissue, one of LGIN, three of HGIN, and three of HSCC. Subsequently, we classified the study categories into four groups based on pathological grading: Normal, LGIN, HGIN, and Tumor (7, 8).

### 2.2 Preparation of single cell suspensions

After sampling, tissues were rinsed of any blood stains with saline and stored in brown tubes with MACS^®^ Tissue Storage Solution (Miltenyi Biotec), transported to the laboratory at 4°C. Dissociation experiments began within 1 hour of arrival. Samples were segmented into 2-3 mm pieces and processed to single-cell suspensions using the Human Tumor Dissociation Kit protocol. Tissue pieces were transferred to a 5 mL tube with dissociation solution and dissociated at 37°C for 2 hours using a rotary mixer. Post-dissociation, 20 mL of DMEM was added, and the suspension was filtered using a 70 μm strainer. Cells were collected by centrifugation at 4°C. Cells were then resuspended in 1×PBS and treated with Red Blood Cell Lysis Solution. Finally, cells were resuspended in 1×PBS + 0.04% BSA + 1U/μL RNase inhibitor. Cell viability, concentration, and aggregation rate were measured using AO/PI Fluorescent Dye on a LUNA-FL™ Cell Counter, with required standards of viability >75%, concentration between 700-1200 cells/μL, and aggregation rate <5%.

### 2.3 Library construction and Next generation sequencing

We utilized Chromium Next GEM Single Cell 5’ Reagent Kits v2 (Dual Index) from 10× Genomics to construct single-cell libraries, aiming for 10,000 captured cells per sample. After generating GEMs with the Chromium Controller, we adhered to the kit instructions for 5’ single-cell RT-PCR amplification, 5’ cDNA amplification and purification, TCR and BCR sequence amplification and purification, and library construction.

The quality and concentration of the library were assessed using a Qubit 4.0 and Qubit™ 1× dsDNA Assay Kits (high sensitivity) from Thermo Fisher Scientific. The molar concentration and fragment insertion of the library were evaluated using the StepOnePlus™ Real-Time PCR System from Applied Biosystems and detected by LabChip Touch. Sequencing was executed on Illumina’s Novaseq 6000 with a PE150 read length.

For processing single-cell 5’ gene expression and TCR enrichment data, we employed Cell Ranger count and Cell Ranger vdj functions of Cellranger software (version 6.0.1) from 10× Genomics. Gene expression data were aligned to the human genome reference (GRCh38), and TCR and BCR enrichment data to the VDJ reference sequences available at 10× Genomics Reference Data.

### 2.4 Single-cell gene expression quantification and determination of cell types

We processed the sequencing data utilizing the Seurat R package (version 4.3.0) (9). Data was converted into a Seurat object and quality filtered to exclude cells meeting specific conditions. Perform batch effect correction using the harmony package (version 1.0.3). Logarithmic normalization and linear regression were performed using Seurat package functions to construct the gene expression matrix. The COSG package (version 0.9.3) identified cell clusters, categorized into six primary cell types based on distinct marker genes: T cells, myeloid cells, B cells, epithelial cells, mast cells, and fibroblasts (10).

Subsequently, normalization, scaling, and clustering were repeated to further subdivide and label specific cell subtypes based on average expression of gene sets in each primary cell type. Cells with multiple labeled genes and elevated UMI counts were considered cellular contamination and excluded. Each cluster of a primary cell type was assigned a cluster identifier containing a marker gene, selected based on criteria including top ranking in differential gene expression analysis, high specificity of gene expression, and literature support validating their role as marker genes or functional genes associated with the cell type.

### 2.5 Pathway enrichment analysis

We used the clusterProfiler software package (11) (version 4.0.5) for R for Gene Ontology (GO), KEGG Pathway, and Reactome enrichment analyses to explore the functions and mechanisms of the identified cellular clusters. P-values were adjusted with the Benjamini and Hochberg method, with p.adjust values below 0.05 deemed statistically significant. Additionally, we performed Gene Set Variation Analysis (GSVA) using the GSEABase package (12) (version 1.62.0) for R, primarily focusing on the 50 hallmark gene sets from the MSigDB database (https://www.gsea-msigdb.org/).

### 2.6 CNV estimation

To identify malignant cells in HSCC patients, we used inferCNV software (version 1.14.2) to infer CNVs from chromosomal gene expression patterns, setting a cutoff value at 0.1 and enabling the denoise option. We chose the expression profiles of T and B cells as references, including all epithelial cell clusters in the observation group. We then evaluated the CNVs signal intensity of each cell by scaling its corrected expression from -2 to 2, establishing the CNV score as the aggregate of the scale scores for each cell. By integrating the sample sources of cell clusters and performing statistical tests between cluster CNV scores, we determined which cells were malignant.

### 2.7 Tissue distribution of clusters

To evaluate the tissue preference of each cell population, we computed the ratio of the number of observed cells to the expected number of cells (Ro/e) across different tissues, allowing statistical inference of cell population preference for specific tissues (13, 14). We used a chi-square test to determine the expected number of cells for each combination of cell population and tissue, offering a robust statistical basis to validate observed distributions against expected ones.

### 2.8 Immunofluorescence staining

The Human Protein Atlas (HPA) database was employed to gather comprehensive information on the human protein, matrix metalloproteinase 3 (MMP3). The HPA database provides a rich resource of data, including immunofluorescence staining results, enabling the exploration of the spatial distribution and expression levels of proteins across different tissues and cells.

### 2.9 Developmental trajectory inference

Initially, we used the CytoTRACE package (15) (version 0.3.3) to infer evolutionary relationships among single cells by assigning an evolutionary score to each cell based on scRNA-seq data, revealing potential evolutionary connections between cells. Next, we used the Monocle3 package (16) (version 1.3.1) to infer evolutionary trajectories, identify cell states, and visualize transition events, enhancing understanding of cellular development and transitions. Finally, to fully understand evolutionary genes and pathways, we used the ClusterGVis package (version 0.1.0) for displaying evolutionary genes and conducting pathway enrichment analysis, integrating diverse data sources and providing extensive visualization options to understand the evolutionary dynamics within cellular populations better.

### 2.10 Survival analysis with TCGA data

We analyzed bulk RNA-seq data from >500 head and neck squamous cell carcinoma (HNSCC) samples profiled by The Cancer Genome Atlas (TCGA) using GEPIA2 (17) and performed Kaplan-Meier analysis. Patients were divided into high and low expression groups based on median gene expression; the top 50% were high expression, and the bottom 50% were low expression. Survival curves were compared using the Log-rank test. GEPIA2 also enabled pan-cancer expression analysis and visualization of MMP3 expression across different cancers.

### 2.11 TCR/BCR repertoire analysis

We utilized the immunarch package (version 0.9.0) for extensive analysis of TCR-βchain and BCR in the dataset. This package offers specialized functions for immunoreceptor analysis, which were employed for all data processing and analysis steps, including TCR and BCR sequence identification, clustering, and diversity analysis, using the package’s default parameters to maintain accuracy and reproducibility.

### 2.12 qRT-PCR analysis

We chose five patients with untreated HSCC, confirmed pathologically, from the Cancer Hospital of the Chinese Academy of Medical Sciences between January 2016 and May 2017. Biopsies of cancerous and adjacent normal tissues were performed on each patient. The patients had a median age of 61, with detailed characteristics available in **Supplementary Table 3**. All participants provided written informed consent, and the study received approval from the hospital’s Ethics Committee.

RNA from the samples was extracted using Invitrogen reagents and converted to cDNA using the High-Capacity cDNA Reverse Transcription Kit from Applied Biosystems. qRT-PCR analysis was performed with Invitrogen qPCR reagents, and further PCR analysis was conducted using the Agilent cDNA Reverse Transcription Kit, with an Agilent (Stratagene MX3005p) device. Specific primers were used for MMP3, MMP7, MAGEA3, and GAPDH, which served as an internal reference (**Supplementary Table 2**). All procedures were replicated three times, and the ΔΔCt method was used to calculate relative gene expression levels.

### 2.13 Analysis of intercellular communication

The CellChat package in R (version 1.6.1) has been developed for inferring and analyzing intercellular communication networks from scRNA-seq data through network analysis, pattern recognition, and various learning methods (18).

### 2.14 Statistics analysis

All statistical analyses were executed with R software, utilizing tests such as the two-sided paired Student’s t-test, two-sided Wilcoxon test, two-sided Pearson correlation test, and two-sided Kruskal-Wallis test. A p-value below 0.05 was deemed to signify a statistically significant difference.

## 3 Results

### 3.1 Landscape view of cell composition in HSCC and its precancerous lesions

**Figure 1:**
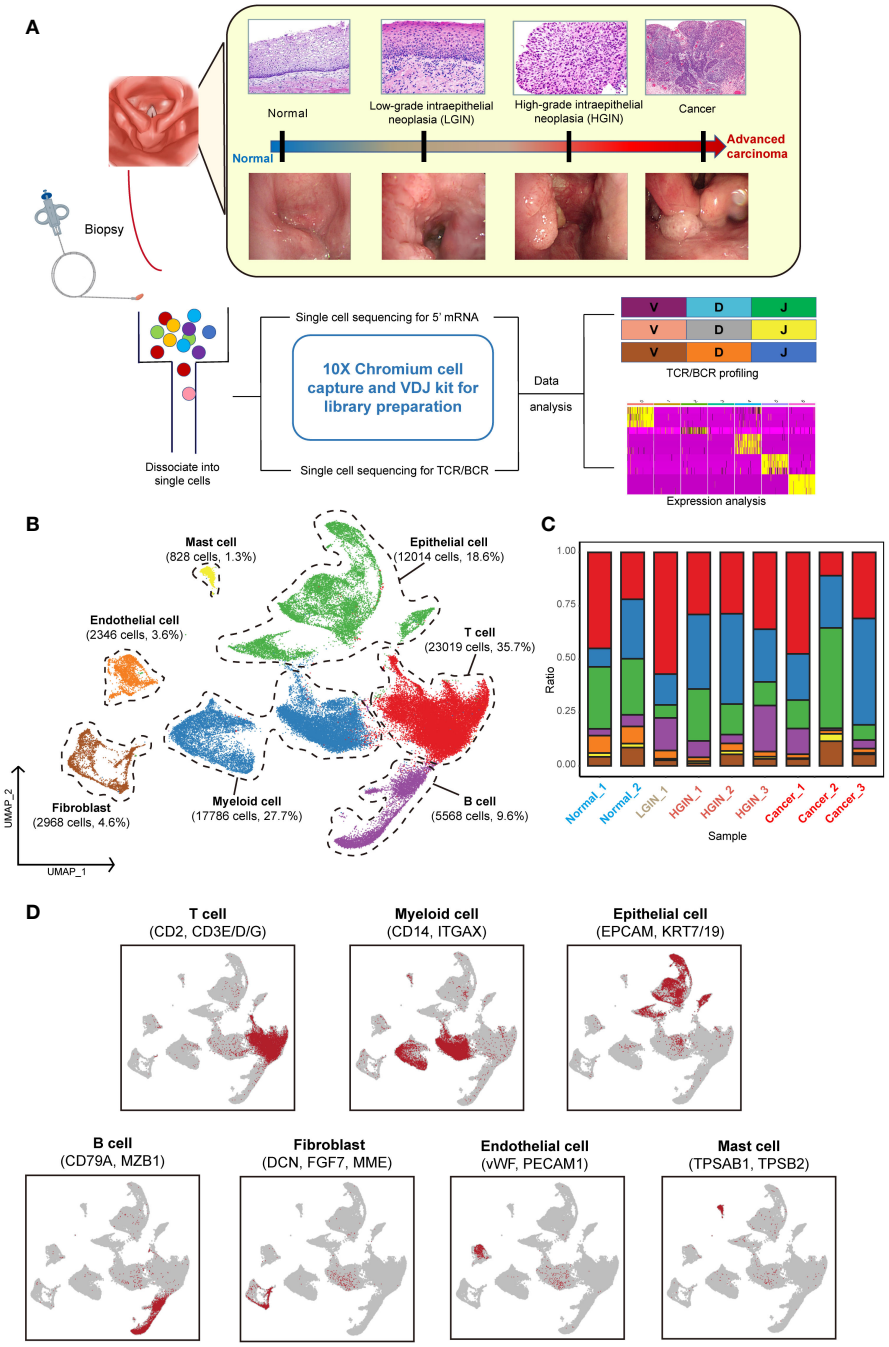
Single-cell landscape profiling across various stages of hypopharyngeal carcinogenesis. A schematic diagram depicts the workflow for sample selection and analysis of HSCC and its precancerous lesions **(A)**. A UMAP diagram displays the annotated cell clusters from our study **(B)**. A bar chart illustrates the proportion of cell types present in each individual based on the scRNA-seq data **(C)**. The expression levels of distinct marker genes across the annotated cell types are presented **(D)**.

To explore the TME during hypopharyngeal carcinogenesis in detail, we performed 5’ RNA sequencing and constructed TCR and BCR libraries on various tumor tissue samples, including the adjacent normal tissue, LGIN, HGIN, and HSCC, using scRNA-seq technology (**Supplementary Tables 1, 2**). Subsequent TCR/BCR analysis and expression profiling were conducted (**Figure 1A**).

To minimize individual variances, we selected patients with two or more different lesion stages, including normal tissue. Each sample yielded an average of over 290 million sequencing reads, with an average sequencing saturation of 88.01% (**Supplementary Table 1**). After stringent quality control, we analyzed a total of 64,529 cells across the samples, identifying approximately 2,224 genes and 9,117 UMIs per cell (**Supplementary Figure 1A**).

For identifying diverse cell types, we used the Seurat package for unsupervised cluster analysis, identifying 12 clusters (**Supplementary Figures 1B, C**). These were classified into seven distinct cell types using classical markers: T cells, myeloid cells, epithelial cells, B cells, endothelial cells, mast cells, and fibroblasts (**Figures 1B, D**) (19). T cells, myeloid cells, and epithelial cells constituted the majority of the total cell population, over 72%, with immune cells representing over 75% of all cells. The presence of cells from multiple samples within the same clusters indicated minimal batch effect (**Figure 1C**; **Supplementary Figures 1B, F**).

During the LGIN stage of HSCC, there is a notable increase in the proportion of T cells, suggesting potential extensive immune stimulation at this stage. Myeloid cells show consistent augmentation post lesion onset. The most significant alterations across stages are seen in immune and epithelial cells (**Supplementary Figure 1D**). Endothelial cells are primarily found in normal tissue, whereas fibroblasts are more prevalent in tumor tissues (**Supplementary Figure 1E**).

### 3.2 The dynamic multidimensional features of epithelial cells in the development of HSCC

**Figure 2:**
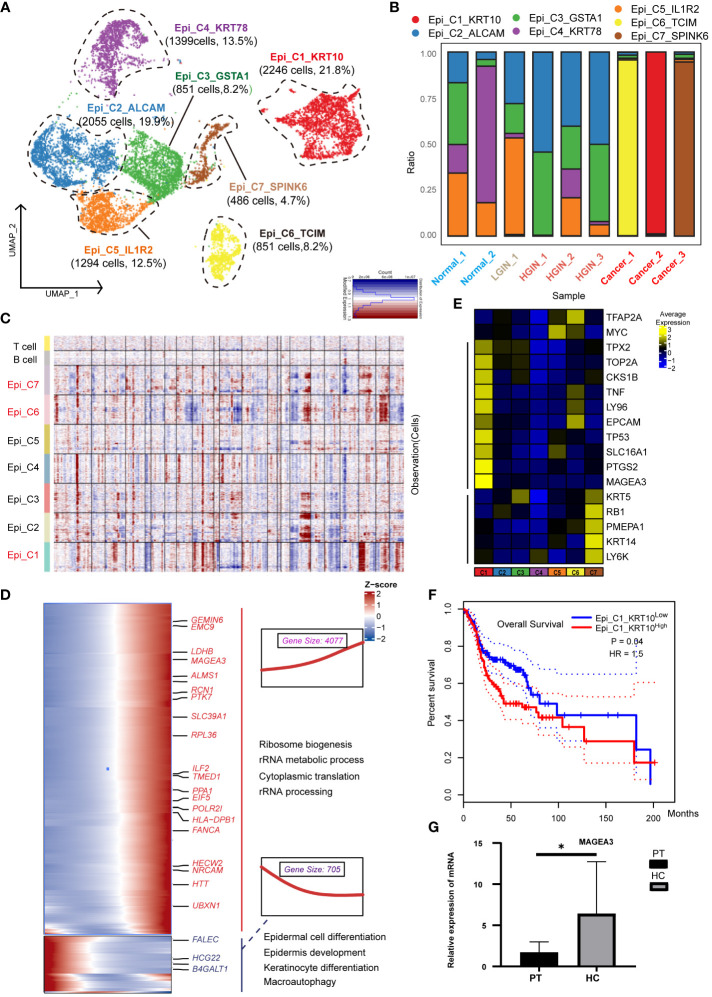
Heterogeneity of Epithelial Cells Across Different HSCC Lesion Stages. A dot plot illustrated the subclusters of epithelial cells across various HSCC lesion stages **(A)**. A bar graph depicted the proportion of cell types within each epithelial cell cluster **(B)**. A heatmap displayed large-scale CNVs for epithelial cells from 9 samples, with CNVs being inferred based on the average expression of 100 genes for each chromosomal position. Gains were indicated in red, and losses in blue **(C)**. Gene expression data was visualized as a heatmap, where high expression was encoded in yellow, and low expression in blue, relative to the mean expression (black) **(D)**. Another heatmap presented different blocks of DEGs along the pseudotime trajectory, with selected KEGG pathways related to the corresponding DEGs on the right. The box highlighted the expression trend and the number of genes in each cluster, showcasing Epi_C4_KRT78 on the left and Epi_C1_SPARC on the right **(E)**. Kaplan-Meier survival curves for HNSCC from The Cancer Genome Atlas were displayed. Intratumoral heterogeneity, based on detected signals for alternative subtypes, was categorized into groups with high and low Epi_C1_KRT **(F)**. The relative mRNA expression of MAGEA3 in paracancerous and HSCC tissues was analyzed using qRT-PCR, where PT represented paracancerous tissue, HSCC represented hypopharyngeal cancer tissue, * represented p-value less than 0.05 **(G)**.

We categorized 12,113 epithelial cells into seven distinct clusters based on expression profiles (**Figure 2A**). Three clusters (Epi_C1_SPARC, Epi_C6_TCIM, Epi_C7_SPINK6) originated from different tumor tissue samples (**Figure 2B**). Given the spectrum of our biopsy samples, transcriptomic profiles might exhibit similarities during the LGIN and HGIN stages, with full heterogeneity manifested among different HSCC patients within tumor tissues (20). We analyzed chromosome copy number variants (CNVs) to delineate tumor cells within these clusters. Several cells from normal samples exhibited CNVs, possibly due to algorithmic properties, sample collection methodologies, or cell post-processing. However, three clusters from tumor cells displayed consistent chromosomal variation (**Figure 2C**). The CNVs scores further illustrated significant differences between these three clusters and the others (**Supplementary Figure 2A**). We identified recognized cancer driver genes (EGFR, MTOR, CCND1, and MYC) within Epi_C1_SPARC and Epi_C6_TCIM subclusters, observing high variability of CNVs in cell proliferation-related genes (TOP2A, MKI67) (**Supplementary Figure 2B**). Epi_C1_SPARC, Epi_C6_TCIM, and Epi_C7_SPINK6 demonstrated elevated expression of tumor and proliferation-related genes, indicating the high proliferative potential and active cytokinesis of the tumor cells (**Figure 2D**).

To understand the transformation from normal epithelial cells to tumor cells, we charted the cell differentiation trajectory from Epi_C4_KRT78 to Epi_C1_SPARC (**Supplementary Figure 2C**) and illustrated gene alterations via a heatmap (**Figure 2E**). The expression of tumor-associated genes like PTK7 and MAGEA3 escalated, implying a potential increase in metabolic activities and nuclear functions during cellular transformation, correlating with elevated proliferation and enhanced viability in tumor cells. Conversely, the expression of genes like FLEC and HGC22 diminished, reflecting a potential loss of normal epithelial cell functions, correlating with abnormal growth and differentiation in tumor cells.

Using data from 523 patients with HNSCC from TCGA, we found a significant correlation between the expression of the Epi_C1_SPARC cluster in tumor tissues and overall survival (OS); higher expression was indicative of poorer prognosis (**Figure 2F**). This implied that this cluster could be a specific marker and a potential therapeutic target for HSCC. When we conducted independent prognostic analysis on its markers, we found that HMGA2 is significantly associated with prognosis and shows great potential in immunotherapy (**Supplementary Figure 3**;**Supplementary Data 1**). In various cancers such as nasopharyngeal carcinoma and lung cancer, HMGA2 is often overexpressed or undergoes gene mutations, which are correlated with cell transformation, tumor proliferation, invasion, and metastasis (21, 22). Validation of the differentially expressed genes (DEGs) of Epi_C1 through qRT-PCR confirmed significant expression of MAGEA3 in HSCC tissues (**Figure 2G**). MAGEA3 is a known tumor-associated antigen and is considered a potential therapeutic target for various tumors (22).

### 3.3 The dynamic multidimensional features of T cells and the diversity of TCR in the development of HSCC

**Figure 3:**
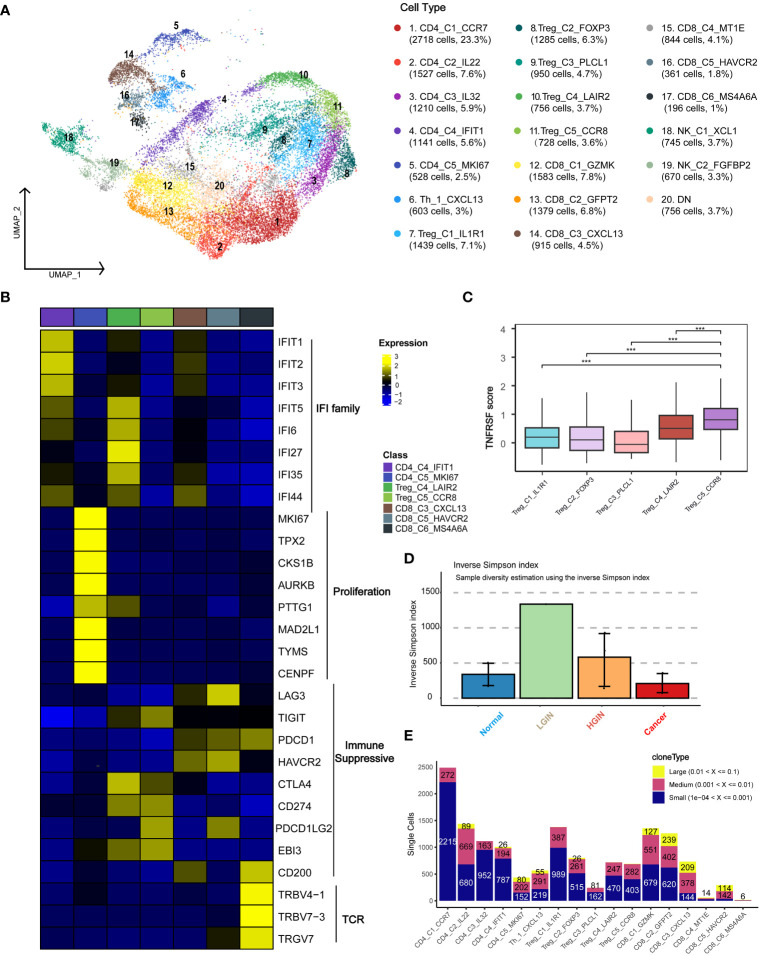
T Cell Heterogeneity Across Different HSCC Lesion Stages. A dot plot illustrated the subclusters of T cells across various HSCC lesion stages **(A)**. Gene expression data was visualized as a heatmap, where high expression was encoded in yellow, and low expression in blue, relative to the mean expression (black) **(B)**. Box plots depicted the TNFRSF scores across different Treg subgroups, including TNFRSF4/8/9/18 **(C)**. A bar plot showcased clonotype diversity in samples from different HSCC lesion stages, calculated using the Inverse Simpson index method **(D)**. Another bar plot presented the counts of clone type frequency groups within different T cell clusters **(E)**. *** represents p<0.001

To investigate the presence and anti-tumor functions of T and NK cells at different HSCC stages, we examined the functional subtypes of these cells and analyzed corresponding alterations in TCR. We segregated 23,019 T and NK cells into 20 distinct subgroups, primarily encompassing CD4+ and CD8+ T cells (**Figure 3A**; **Supplementary Figure 4A**). Across all samples of HSCC, we pinpointed a total of 5,278 T/NK cells. Based on the expression of markers, we organized them into 20 clusters, which included: five CD4 clusters (two Naïve clusters: CD4_C1_CCR7 and CD4_C3_IL32, one highly proliferative cluster: CD4_C5_MKI67, one Th cell: Th_1_CXCL13, and five Treg clusters: Treg_C1_IL1R1, Treg_C2_FOXP3, Treg_C3_PLCL1, Treg_C4_LAIR2, Treg_C5_CCR8); six CD8 clusters (three exhausted T-cell clusters: CD8_C3_CXCL13, CD8_C5_HAVCR2, CD8_C6_MS4A6A, and one effector memory cluster: CD8_C1_GZMK); and NK cell subsets, categorized into resting NK cells: NK_C1_XCL1 and cytotoxic NK cells: NK_C2_FGFBP2.

Initially, we categorized the cells into CD4+ T cells, CD8+ T cells, and double-negative cells, based on the expression levels of CD4 and CD8 genes. CD4_C1_CCR7 and CD4_C3_IL32 were identified as Naive T cells due to the expression of characteristic markers such as CCR7, SELL, and CD27. Th_1_CXCL13 was identified as a typical Th1 cell, expressing CXCL13, BHLHE40, and CXCR3. Within the immunoprofile, we discerned five Treg clusters, expressing not only Treg markers like FOXP3, TNFRSF18, and TNFRSF4 but also various exhaustion markers including LAG3, TIGIT, PDCD1, HAVCR2, and CTLA4. CD8_C4_IFIT1 and Treg_C4_LAIR2 predominantly originated from tumor samples, while CD8_C3_CXCL13 and CD8_C6_MS4A6A were primarily derived from both tumor and HGIN samples (**Supplementary Figure 4C**).

To identify uniquely expressed genes in specific cell types, we performed a DEGs analysis on T cell clusters and presented the results using a heatmap (**Figure 3B**). The tumor-derived cluster CD8_C4_IFIT1 showed expression of genes related to the IFIT and IFI families, which were crucial for antiviral responses and were closely related to interferon immune responses and immune microenvironment regulation (23).

The CD4_C5_MKI67 cluster was highly proliferative, suggesting active cell division and a positive response to tumor antigen-specific stimuli, making them potential immunotherapy targets. Treg cells, crucial for mitigating immune responses and maintaining immune homeostasis (24), predominantly expressed key immunosuppressive molecules like CTLA-4, PDL1, and TIGIT.

Exhausted T cells such as CD8_C3_CXCL13 and CD8_C5_HAVCR2 expressed markers including LAG3, PDCD1, and HAVCR2. We also identified a cluster, CD8_C6_MS4A6A, related to antigen recognition activation and T cell clonal proliferation. The Treg_C5_CCR8 cell cluster showed significant immunosuppressive functionality due to elevated expression of TNFRSF family genes and CCR8, facilitating immune evasion and cancer progression (**Figure 3C**). Disrupting CCR8 signaling could potentially inhibit tumor growth by attenuating the suppression of cytotoxic lymphocytes (25).

We examined TCR α-chain and β-chain sequences, identifying 19,365 T cells or clonotypes with TCR α-β pairings (**Supplementary Table 3**). Clonotypes in T cells showed an ascending trend from normal samples to LGIN (**Figure 3D**). In exploring T cell clonality in HSCC’s TME, we observed signs of clonal expansion in tumor-infiltrating T lymphocytes (**Figure 3E**). Interestingly, no identical TCR clonotypes were identified among most samples (**Supplementary Figure 4B**), with identical ones observed only in samples from the same patient at different lesion stages (**Supplementary Figure 4D**). One of the most variable shared CDR3 sequences had partial associations with the Epstein-Barr virus and cytomegalovirus (**Supplementary Table 2**). We also discovered that the majority of TCRs detectable in T cells is TRB, with 82.7% of T cells exhibiting TCR expression. TCR expression is present across most cell cluster, however, it was also observed that NK cell clusters, NK_C1_XCL1 and NK_C2_FGFBP2, rarely express TCR. Additionally, CD8_C5_HAVCR2 and CD8_C6_MS4A6A show increased TCR expression during the HGIN and tumor stages. Overall, the trend indicates that TCR variations in T cells intensify with the progression of the lesion, yet a significant functional subcluster transition occurs during the tumor stage (**Supplementary Figure 4E**).

### 3.4 The dynamic multidimensional features of B cells and the diversity of BCR in the development of HSCC

**Figure 4:**
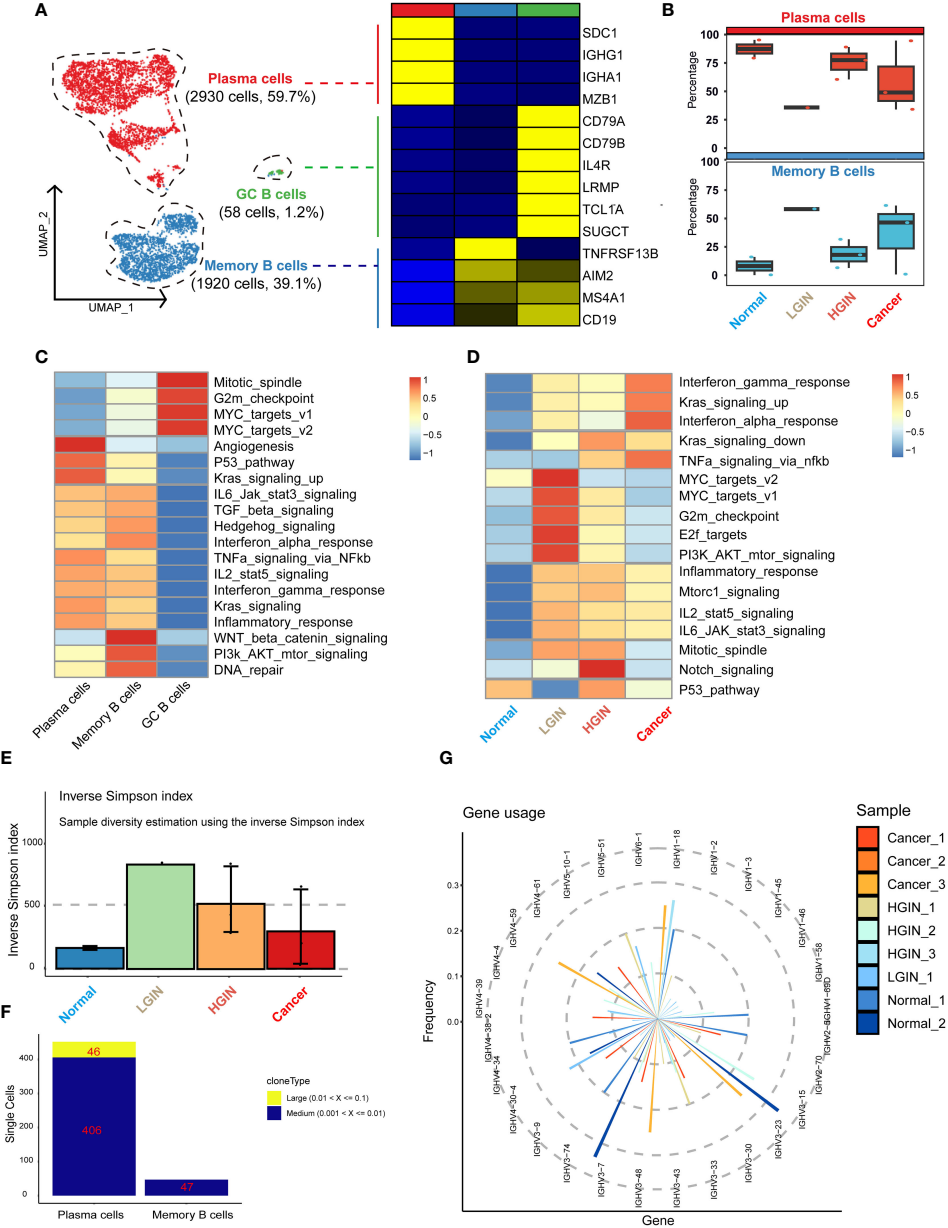
B cell heterogeneity in different HSCC lesion stages. The UMAP on the left showed the B-cell clusters present in different HSCC lesion stages, and the gene expression data on the right were displayed as a heatmap, with high expression in yellow and low expression in blue relative to the average expression (black) **(A)**. Box plots suggested cell occupancy in plasma cells versus memory B cells in different HSCC lesion stages **(B)**. A heatmap showed scaled GSVA enrichment scores for pathways associated with tumors among different B cell subclusters **(C)**. Another heatmap displayed scaled GSVA enrichment scores for pathways associated with tumors in plasma cells in different HSCC lesion stages **(D)**. A bar plot showed clonotype diversity in different HSCC lesion stages, calculated using the Inverse Simpson index method **(E)**. Another bar plot displayed the counts of clone type frequency groups in different clusters of B cells **(F)**. Analysis of IGH gene fragment usage in different HSCC lesion stages was presented **(G)**.

We identified a total of 4,908 B cells and classified them into three distinct clusters: plasma cells, memory B cells, and germinal center (GC) cells. The marker genes for plasma cells included MZB1, SDC1, IGHG1, and IGHA1; for memory B cells included TNFRSF13B, AIM2, MS4A1, and CD19; and for GC cells included CD79A, CD79B, IL4R, LRMP, TCL1A, and SUGCT (**Figure 4A**). As hypopharyngeal tissues progressed to a diseased state, memory B-cell and T-cell subsets showed a substantial increase from normal tissue to LGIN, while plasma cells experienced a significant proportional decrease (**Figure 4A**).

We subsequently performed GSVA pathway enrichment analysis on the three B cell clusters. The analysis showed that GC B cells were primarily enriched in pathways associated with cell proliferation and growth, including Mitotic_spindle and G2m_checkpoint. Plasma cells and memory B cells shared similarities in multiple pathways, but plasma cells had a stronger association with angiogenesis and prominently expressed pathways linked to cancer cell proliferation and growth, such as P53_pathway and Kras_signaling_up. Conversely, memory B cells had a stronger relation to WNT beta catenin_signaling, PI3k AKT_mtor_signaling, and DNA_repair pathways (**Figure 4B**). Analysis of their differential genes revealed that plasma cells mainly enriched in processes ensuring correct antibody folding and modification in the endoplasmic reticulum, including unfolded protein response, translation, and protein folding. Memory B cells predominantly enriched in antigen receptor-mediated signaling pathway, leukocyte proliferation, and interferon gamma signaling, highlighting their capability to swiftly respond to re-encountered antigens and collaborate with other immune cells for crucial immune regulation (**Supplementary Figures 5A, B**).

GSVA pathway enrichment analysis demonstrated that GC B cells primarily enriched in pathways associated with cell proliferation and growth, plasma cells in angiogenesis and cancer cell proliferation, and memory B cells in WNT beta catenin signaling, PI3k AKT mtor signaling, and DNA repair pathways, reflecting their distinct roles in immune regulation, antigen response, and collaboration with other immune cells.

Subsequently, we analyzed plasma cells at different stages through GSVA pathway enrichment analysis. Certain pathways, notably interferon gamma_response, Kras_signaling_up, and interferon alpha_response, displayed consistently high activity (**Figure 4C**), suggesting a dynamic and evolving immune system response in HSCC lesions. The immune response appears to escalate its counteraction against the tumor with the progression of the lesion. In the LGIN stage, four pathways in the plasma cell cluster—MYC_targets_v2, MYC_targets_v1, G2m_checkpoint, and E2f_targets—reached peak activity (**Figure 4D**). Here, plasma cells seemed to be combating initial tumor development, but as the lesion advances, these efforts appeared insufficient to curb the ongoing tumor growth.

Additionally, we examined the BCR library for IGH and IGL chain sequences of the B-cell receptor. Post stringent screening, we identified 5,282 B cells with BCR IGH-IGL pairs or clonotypes (**Supplementary Table 1**). A significant increase in specific clonotypes from normal samples to LGINs was observed (**Figure 4E**), implying that such clonal expansion could be a specialized tumor response. The degree and pattern of clonal expansion differed across cell clusters, with plasma cells showing more clonal expansion than memory B cells, which were pivotal in tumor response and whose expansion might correlate with the generation of specific anti-tumor antibodies (**Figure 4F**). This is consistent with prior observations. We found no identical BCR clonotypes between most samples (**Supplementary Figure 5C**). The CDR3 length in IGH mainly varied from 12 to 22 bases, and in IGL from 11 to 16 bases (**Supplementary Figure 5D**). The frequency and count of IGH-V genes decreased with lesion onset and progression, indicating enhanced B-cell specific responses to certain antigens. This implies selective amplification of some V genes and reduction of others, resulting in a decreased overall frequency and count of V genes utilized (**Figure 4G**). We also found that 81.7% of B cells express BCR. Plasma cells significantly overexpress IGH, while memory B cells predominantly express IGK. There are significant differences in BCR expression among different B cell subclusters. Overall, the trend indicates that plasma cells and memory B cells are continuously activated with the occurrence of lesions, leading to an enhanced immune response (**Supplementary Figure 5E**).

### 3.5 The dynamic multidimensional features of myeloid cells in the development of HSCC

**Figure 5:**
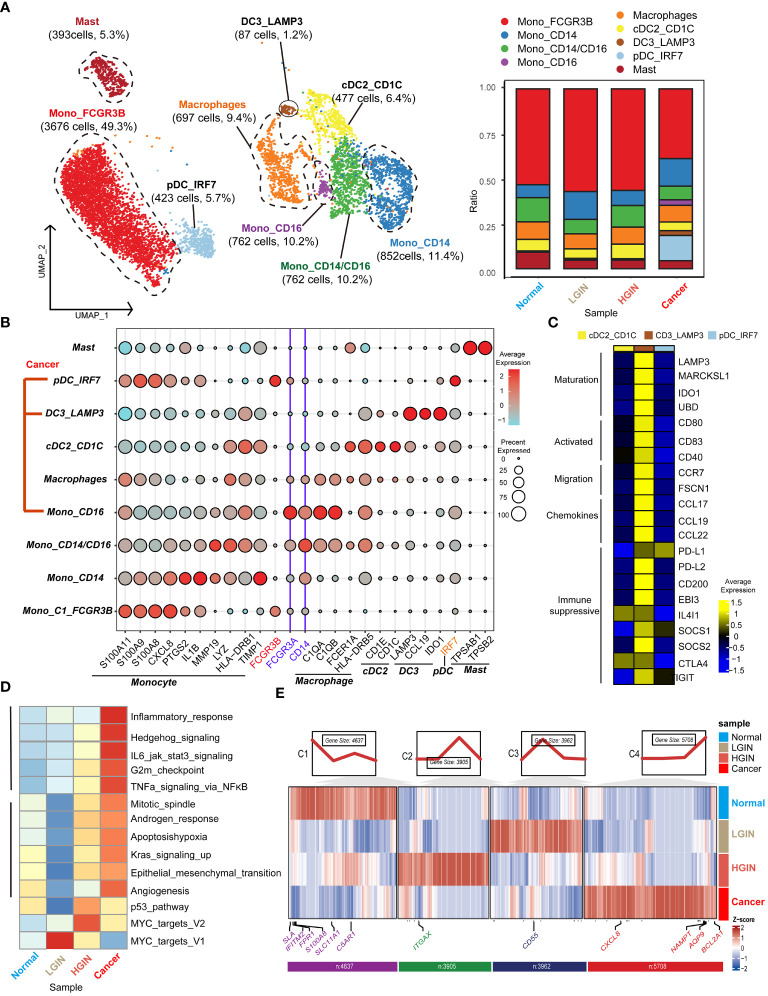
Myeloid cell heterogeneity in different HSCC lesion stages. The UMAP on the left showed the myeloid cell clusters present in different HSCC lesion stages, and the bar graph showed the proportion of cell types contained in each epithelial cell cluster **(A)**. Feature plots of canonical marker genes were displayed **(B)**. The gene expression data on the right were shown as a heatmap, with high expression in yellow and low expression in blue relative to the average expression (black) **(C)**. Box plots suggested cell occupancy in plasma cells versus memory B cells in different HSCC lesion stages **(B)**. A heatmap showed scaled GSVA enrichment scores for pathways associated with tumors among different B cell subclusters **(C)**. Another heatmap displayed scaled GSVA enrichment scores for pathways associated with tumors in Mono_FCGR3B in different HSCC lesion stages **(D)**. A further heatmap depicted DEGs in different HSCC lesion stages in the Mono_FCGR3B cluster; boxes showed expression trends and the number of genes in the cluster **(E)**.

In the TME of HSCC, we identified 12,614 myeloid cells. After annotation, we outlined four monocyte clusters: the prevalent Mono_FCGR3B, the classical double-positive Mono_CD14/CD16, and the single-positive Mono_CD14 and Mono_CD16. We also identified three classical dendritic cell clusters: cDC2_CD1C, DC3_LAMP3, and pDC_IRF7. The distribution of these clusters was generally stable across lesion stages, except for tumor-derived pDC_IRF7, DC3_LAMP3, and Mono_CD16 clusters (**Figure 5A**).

Monocytes, characterized by genes like S100A11, S100A9, CXCL8, and LYZ, were subdivided based on selective expression of CD14 and CD16 (**Figure 5B**). For dendritic cells, emphasis was on the DC3 cluster, recognizing three main clusters marked by genes such as LAMP3, CCL19, and IDO1 (**Figure 5C**). This cluster exhibited genes related to high mutation (e.g., LAMP3, MARCKSL1, IDO1, UBD), migration (e.g., CCR7, FSCN1), and immunosuppression (e.g., PD-L1, PD-L2, CD200, SOCS1, SOCS2, CTLA4, IGIT).

Next, we concentrated on the predominant Mono_FCGR3B cluster within myeloid cells. In the cancer stage, this cluster showed elevated expression in cancer-related pathways like inflammatory response, Hedgehog signaling, and IL6 jak stat3 signaling. Notably, in the LGIN stage, some functions such as mitotic spindle, apoptosis, hypoxia, and angiogenesis, saw a significant decline (**Figure 5D**). In FCRG3B_Mono, we classified gene expression trends into four categories based on the lesion progression of HSCC. In the normal tissue stage, monocytes execute immune surveillance, responding to potential pathogens or injury signals, marked by elevated expression of SLA, FPR1, SLC11A1, and C5AR1. In the LGIN stage, tissues control excessive inflammatory responses, indicated by elevated expression of CD55. In the HGIN stage, monocytes respond to increased inflammatory signals and activate in TME, indicated by elevated expression of ITGAX. In the tumor tissue stage, monocytes remain active in the TME, interacting with tumor cells and other immune cells, marked by elevated expression of CXCL8, NAMPT, AQP9, and BCL2A1 (**Figure 5E**).

### 3.6 The dynamic multidimensional features of endothelial cells in the development of HSCC

**Figure 6:**
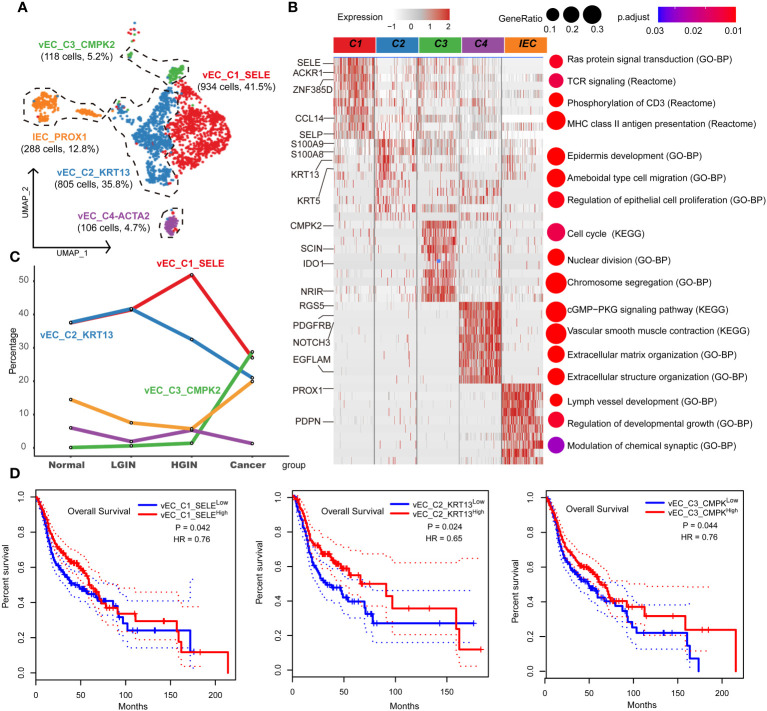
Endothelial cell heterogeneity in different HSCC lesion stages. The UMAP on the left showed the endothelial cell clusters present in different HSCC lesion stages **(A)**. The heatmap on the left displayed the differentially expressed genes (DEGs) of different endocytic clusters, and the GO-BP, KEGG, Reactome enrichment results of each cluster were shown on the right **(B)**. A line graph depicted the change in the percentage of cell clusters in different lesion types **(C)**. Kaplan-Meier survival curves were displayed for HNSCC from the Cancer Genome Atlas. Intratumoral heterogeneity was estimated based on detected signals for alternative subtypes and was divided into two groups with high and low vEC_C1-C3 **(D)**.

Endothelial cells were integral to the TME in HSCC, crucially influencing tumor angiogenesis, growth, and metastasis. Investigating these cells provided insights into the interactions and functions between the TME and tumor cells. After rigorous quality control, we classified 2,346 cells into five endothelial cell clusters, including four vascular (vEC_C1_SELE, vEC_C2_KRT13, vEC_C3_CMPK2, vEC_C4_ACTA2) and one lymphatic endothelial cell (IEC_PROX1) (**Figure 6A**). FLT1 marked vascular endothelial cells, while PROX1 and CCL21 marked lymphatic ones.

Tracking lesion progression, we discerned three distinct cell clusters. The proportion of vEC_C1_SELE increased from normal to HGIN stage but decreased in the tumor stage. Enrichment analysis of this cluster indicated Ras protein signaling and MHC II antigen presentation, implying enhanced cell signaling and antigen presentation in early-stage lesions to bolster immune response. However, in the tumor stage, these cells’ functions might have been suppressed or overtaken by other clusters.

vEC_C2_KRT13 demonstrated a decline in the tumor stage. Enrichment analysis associated this cluster with pathways related to cell migration, proliferation, and skin development, highlighting its role in sustaining tissue structure and function in normal tissues. vEC_C3_CMPK2, primarily from tumor samples, was enriched in tumor-related pathways like cell cycle and nuclear division, indicating its active and proliferative state during tumor development (**Figures 6B, C**).

Using TCGA data, we found significant correlations between these three clusters and prognosis. High expression of vEC_C1_SELE and vEC_C2_KRT13 correlated with better prognosis, while low expression of vEC_C3_CMPK2 indicated a good prognosis (**Figure 6D**). The association of low expression of vEC_C3_CMPK2 with a good prognosis may signify its role in facilitating tumor progression, representing tumors in a state of high proliferation and activation, correlating with poor prognosis.

### 3.7 The dynamic multidimensional features of fibroblasts in the development of HSCC

**Figure 7:**
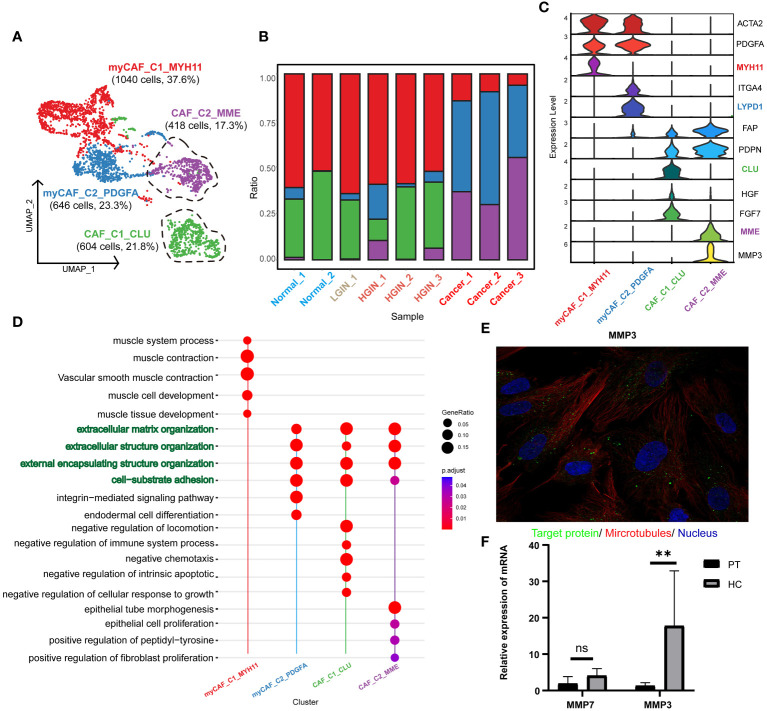
Fibroblast cell heterogeneity in different HSCC lesion stages. The UMAP showed the fibroblast cell clusters present in different HSCC lesion stages **(A)**. The bar graph displayed the proportion of cell types contained in each fibroblast cell cluster. Feature plots of canonical marker genes were presented **(B)**. Violin plots showed the normalized expression of markers (rows) in each fibroblast cell cluster (columns). Cell clusters and the expression level of a gene were indicated at the x- and y-axis, respectively **(C)**. A Dotplot displayed GO-BP, KEGG, Reactome enrichment results for each fibroblast cluster **(D)**. Immunofluorescence showed positive expression of MMP in human fibroblasts. MMP: green; microtubules: red; nucleoli: blue **(E)**. The relative expression of mRNA for MMP3 in the paracancerous and HSCC tissues was analyzed using qRT-PCR, where PT represented paracancerous tissue, HC represented hypopharyngeal cancer tissue, NS represented no significance, and ** represented p-value less than 0.01 **(F)**.

After stringent quality control, we isolated 2968 fibroblasts and identified two primary fibroblast classes through unsupervised clustering: tumor-associated fibroblasts (CAF_C1_CLU, CAF_C2_MME) and tumor-associated myofibroblasts (myCAF_C1_MYH11, myCAF_C2_PDGFA) (**Figure 7A**). CAF was characterized by FAP and PDPN, with ACTA2 and PDGFA distinctive for myCAF. (**Figure 7C**). Analysis showed that CAF_C2_MME and myCAF_C2_PDGFA mainly originated from tumor samples (**Figure 7B**; **Supplementary Figure 6A**).

Our annotations correlated with the results of the pathway enrichment analysis. myCAF_C1_MYH11 was enriched in muscle system processes and muscle contraction pathways, while the others were associated with extracellular matrix organization and structure, aligning with the primary function of fibroblasts in maintaining tissue structure and function. CAF_C1_CLU was enriched in pathways related to negative chemotaxis and intrinsic apoptosis regulation, suggesting a role in inhibiting immune cell migration and enhancing cell survival. CAF_C2_MME was associated with epithelial cell and fibroblast proliferation, implying a role in promoting tumor cell and fibroblast proliferation. myCAF_C1_MYH11 was enriched in muscle contraction pathways, aligning with the function of myofibroblasts in tissue contraction. myCAF_C2_PDGFA was enriched in integrin-mediated signaling pathways, implying a role in tissue remodeling and repair (**Figure 7D**, **Supplementary Figure 6B**).

Focusing on CAF_C2_MME in HSCC, where low expression correlated with better prognosis, we explored its functional genes and identified two key genes: MMP3 and MMP7 (**Supplementary Figures 6C, D**), involved in the degradation and remodeling of the extracellular matrix. Validation through the HPA database and qRT-PCR revealed significant expression of MMP3 in normal human fibroblasts and higher expression in HSCC tissues (**Figures 7E F**). In the TCGA database, MMP3 showed significantly higher expression in HNSCC samples, emphasizing the importance of MMP3 in HSCC (**Supplementary Figure 6E**). We propose that MMP3 is a marker for tumor-associated fibroblasts and a specific biomarker for HSCC.

### 3.8 Communication and interaction between cell subclusters in HSCC

**Figure 8:**
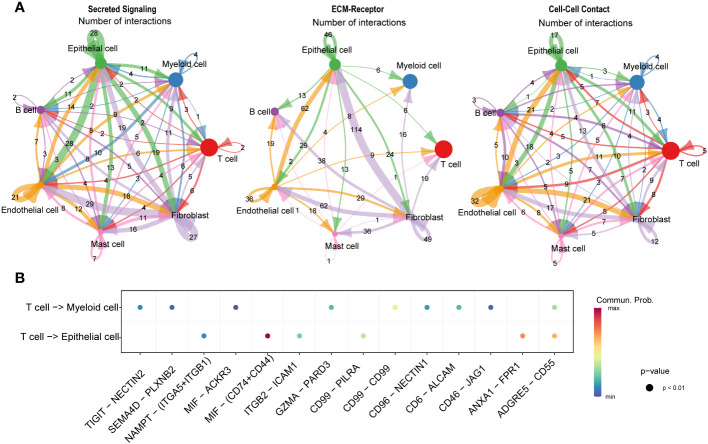
Communication and interaction between cell subclusters in HSCC. Circos plots displaying interactions and connections between cell subpopulations. Divided into Secreted Signaling, ECM-Receptor, and Cell-Cell Contact dimensions. Interactions are divided into incoming and outgoing events. The brand links pairs of interacting cell types, and its width is proportional to the number of events **(A)**. Dot plot showing ligand-receptor analysis between T cells and myeloid and epithelial cells **(B)**.

We conducted an intercellular communication analysis on all cell subpopulations, which was divided into three dimensions. In the secreted signaling dimension, intercellular communication was most abundant, with extensive connections existing between T cells, B cells, epithelial cells, endothelial cells, and fibroblasts. Within the ECM-Receptor interaction, the exchange was most significant among stromal cells such as epithelial cells, endothelial cells, and fibroblasts, including self-regulation mechanisms. In the cell-cell contact dimension, the connection between endothelial cells and epithelial cells was particularly notable (**Figure 8A**). We further revealed the links between T cells, serving as ligands, and epithelial cells as well as myeloid cells, discovering significant ligand-receptor pairs such as MIF-(CD74+CD44) with epithelial cells and MIF-ACKR3 with myeloid cells (**Figure 8B**).

## 4 Discussion

HSCC is the most aggressive subtype of HNSCC. Therefore, exploring the mechanisms of HSCC, especially the transformation from precancerous lesions to tumors, is vital for developing early diagnostics and innovative treatments to enhance survival and life quality for patients. Precancerous lesions, present before cancer onset, can accelerate cancer progression (26). Clinically, patients with HSCC often have visible precancerous lesions in the mucosal layer, appearing as erythematous patches, near areas with visible cancerous changes (27). These lesions can develop into invasive cancers. Traditional sequencing often fails to precisely differentiate the characteristics of diverse cell populations in HSCC and its precancerous stages (28). The introduction of the first scRNA-seq method by Tang et al. allows for detailed examination of cellular composition and molecular characterization, providing insights into transcriptional states at a single-cell level for unbiased cell population analysis (29).

Tumor cells exhibit aneuploidy and chromosomal CNVs, indicative of significant heterogeneity in HSCC cells. This heterogeneity is subtle in initial stages like HGIN, where tumor cells have not undergone extensive mutations and selections (30). However, it becomes pronounced as the disease progresses to the neoplastic stage, due to the accumulation of mutations and adaptations (31). Our observations indicate that during tumorigenesis, tumors experience structured and consistent transcriptional state diversification, with epithelial cells developing new states while preserving the old ones, contrasting the embryonic developmental process where new states are acquired and old ones are discarded (32). These insights suggest that tumor cells disrupt the normal epithelial functional program to acquire new states, which, once established, tend to dominate overwhelmingly in proportion.

The epithelial to tumor transition is characterized by increased proliferation and survival capabilities and loss of normal functions. We identified MAGEA3, a classical oncogene, as significantly upregulated during carcinogenesis, with substantial prognostic significance. MAGEA3, a cancer-testis antigen, is present in various cancers, the testis, and the placenta, but absent in other somatic cells. It interacts with numerous transcription factors and co-regulators, impacting gene expression regulation, and is a reliable target for immunotherapy (33). Clinical trials have demonstrated the therapeutic efficacy of targeting MAGEA3, highlighting its potential as a therapeutic target for HSCC (20).

In our study, immune cells, making up over 75% of cell clusters in HSCC, were central due to their role in cancer development within the TME. The variety and amount of immune cells in this environment significantly affect tumorigenesis and immunotherapy effectiveness. Both intrinsic and adaptive immune cells in the TME contribute to tumorigenesis, highlighting the important interaction between different cell types in cancer progression (34).

CD8+ T cells, essential for antitumor immunity, can destroy tumor cells (35). Our study in HSCC found many tumor-infiltrating CD8+ T cells showing clonal expansion and suppression by tumor neoantigens, aligning with findings in other cancers (36), indicating a universal immunosuppressive state of CD8+ T cells during tumorigenesis.

Integrating immunobanking and transcriptomic analyses, we identified the most significant immunostimulatory response during the transition from normal hypopharyngeal tissue to LGIN, characterized by increased diversity and abundance of TCR and BCR clonotypes. This phase experiences a rise in memory B cells and a decrease in plasma cells. The expansion of memory B cells, capable of a rapid immune response to a known antigen, may represent the body’s effort to combat mutant cells in response to tumor antigens (37). In contrast, the reduction in plasma cells, responsible for antibody secretion, may signify a suppressed sustained immune response, possibly due to immunosuppressive factors released by epithelial cells or the tumor inhibiting normal immune cell function (38).

FCRG3B_Mono, the predominant cell within the myeloid cluster (>49%), maintains stable numerical proportions at the lesion onset but undergoes significant functional changes. In the phase of LGIN, it shows suppressed proliferation and a reduced response to injury, suggesting its immunosuppressive state may aid the progression of HSCC (39). Modulating the immune state of FCRG3B_Mono may offer control over the cancer’s progression (40).

cDC1_CD1C, a crucial DC subtype, initiates anti-tumor CD8+ T cells and is vital for anti-tumor immunity (41). In contrast, DC3_LAMP3 expresses markers of DC activation and attracts naïve T cells and other DCs through CCL19 interaction with CCR7 (42). This suggests DC3_LAMP3 is a regulatory and tolerogenic DC, with high expression of migratory and maturation-related genes, playing a role in immune response modulation within the tumor environment (43).

Endothelial cells play a crucial role in cancer occurrence and treatment. We identified four distinct vascular endothelial cell clusters, three of which have significant prognostic value in HNSCC. The vEC_C1_SELE cluster, enhancing cell signaling and antigen presentation in early lesions, decreases substantially in the cancer stage, suggesting its contribution to inhibiting tumor progression (44). In contrast, the vEC_C2_KRT13 cluster, crucial for maintaining normal vascular structure, shows a continuous decline, indicating a potential correlation with tumor angiogenesis and growth (45). The vEC_C3_CMPK2 cluster is associated with tumor cells and may contribute to the formation of vascular mimicry, facilitating tumor growth and metastasis (46).

Fibroblasts, integral components of the TME, interact with various cells influencing tumor progression (47). They can transform into CAFs, secreting substances that facilitate tumor activities and suppress immune responses (48). Given CAFs’ significant role in tumorigenesis, they are becoming promising therapeutic targets, with strategies focusing on their interactions offering innovative therapeutic approaches (49). In our study, we discovered a unique CAF cluster in HSCC, CAF_C2_MME, originating from tumor samples, with MMP3 as its associated gene, predominantly expressed in fibroblasts and significantly overexpressed in HSCC tissues. The matrix metalloproteinase (MMP) family, including MMP3, influences tumorigenesis by impacting fibroblasts and degrading the extracellular matrix, potentially facilitating tumor cell invasion and migration (50). In HSCC, MMP3 expression may correlate with the activation and aggregation of CAF, impacting tumor progression and prognosis. Thus, MMP3 could be a marker for CAF and a therapeutic target in HSCC. Exploring the role and mechanism of MMP3 may lead to innovative therapies to inhibit tumor invasion and metastasis effectively, enhancing patient survival and quality of life.

We also discovered that T cells interact with the CD74+CD44 complex on epithelial cells and the ACKR3 receptor on myeloid cells through the secretion of MIF (macrophage migration inhibitory factor), revealing a complex and refined communication mechanism between the immune system and other cell types (51). MIF, a critical immune regulatory factor, can modulate inflammatory responses, immune responses, and cell migration and survival across various cell types through binding to different receptors. In interactions with epithelial cells, the binding of MIF to CD74 and CD44 may affect cell proliferation, migration, and functions in pathological states, such as promoting tumor cell growth and metastasis in the tumor microenvironment (52). Conversely, interaction with the ACKR3 receptor on myeloid cells may be involved in regulating the recruitment and migration of immune cells, as well as intercellular signal transmission in diseases like inflammation and cancer (53, 54). These findings not only further confirm the diverse roles of T cells in maintaining immune balance and participating in disease responses but also highlight the importance of intercellular communication in immune surveillance and pathological state regulation, providing critical insights for understanding the complexity of the immune system and developing new therapeutic strategies.

However, our study has its limitations. The limited size of our cohort prevents us from drawing definitive conclusions about the correlation between cellular composition and clinical outcomes. Additionally, we have not considered the spatial distribution of cell clusters, which is crucial given the heterogeneous distribution of immune cells within the tumor. Future research should explore the spatial heterogeneity of the TME in HSCC, potentially employing advanced technologies such as spatial transcriptomics for more comprehensive insights. Another limitation of our study is the lack of experimental validation of the proportions of cell subpopulations within each sample. We have adopted advanced statistical methods and conducted indirect validations through existing datasets. While these methods cannot replace direct validation techniques such as multiplexed immunohistochemistry (mIHC) or fluorescence-activated cell sorting (FACS), they do enhance the reliability of our research findings. We also plan to perform mIHC and FACS validations in our future research efforts and intend to significantly increase our sample size to improve the robustness and generalizability of our results. Lastly, although we have identified and validated key molecules through bioinformatics and qRT-PCR experiments, further functional experiments are necessary to explore their biological significance and the mechanisms involved.

In conclusion, our study has revealed dynamic changes in the TME during the progression of HSCC. We have identified key cells and molecules that drive the progression of HSCC, thereby gaining insights into its underlying mechanisms and paving the way for new therapeutic strategies.

## Data availability statement

The datasets presented in this study can be found in online repositories. The names of the repository/repositories and accession number(s) can be found below: https://ngdc.cncb.ac.cn/gsa-human/ under the accession number HRA005736.

## Ethics statement

The studies involving humans were approved by Ethics committee of the Cancer Hospital of the Chinese Academy of Medical Sciences. The studies were conducted in accordance with the local legislation and institutional requirements. The participants provided their written informed consent to participate in this study.

## Author contributions

CT: Conceptualization, Investigation, Software, Writing – original draft, Writing – review & editing. J-QZ: Conceptualization, Investigation, Project administration, Writing – original draft, Writing – review & editing. ZY: Investigation, Writing – review & editing. L-ZD: Writing – review & editing. M-LW: Writing – review & editing. G-QW: Writing – review & editing. X-GN: Conceptualization, Data curation, Funding acquisition, Project administration, Validation, Writing – original draft, Writing – review & editing.
