# Supplementary material for: Revealing molecular and cellular heterogeneity in hypopharyngeal carcinogenesis through single-cell RNA and TCR/BCR sequencing
Source: Front Immunol. 2024 Apr 24;15:1310376. doi: 10.3389/fimmu.2024.1310376 (PMC11076829; doi:10.3389/fimmu.2024.1310376)
Supplement: Supplementary file 1 [file Presentation_1.pdf]

# *Supplementary Material*

**Supplementary Figure 1 Fundamental insights from single-cell RNA sequencing data.**

Violin plots represent basic single-cell sample metrics post-quality control, including nFeature, nCount, percent mito, and percent hemoglobin (A). An initial dot plot showcases the cell subclusters obtained (B) and their marker gene expression (C). A line graph traces the variation in the percentage of cell clusters across different lesion types (D). A line chart reveals tissue prevalence for each cell type, as estimated by the Ro/e score (E). A dot plot demonstrates the distribution of cell clusters within each lesion stage sample (F).

**Supplementary Figure 2 CNV Inference in Epithelial Cells and Pseudotemporal Trajectory Analysis.**

Box plots highlighted the CNV scores of epithelial cell subclusters in comparison to reference subclusters (T cells, B cells) (A). CNV data was visualized through a heatmap, with gains shown in green and losses in purple, relative to no CNV (white) (B). Cell trajectory analysis of the epithelial cell compartment was presented, calculated using Monocle3. The analysis was rooted in transitional Epi_C4_KRT78 (purple) and differentiated into Epi_C1_KRT10 (yellow) (C).

**Supplementary Figure 3 Analysis of hub genes in the Epi_C1_SPARC cluster.**

The survival curve plot shows the prognostic outcomes of HMGA2 and AGR2 in TCGA-HNSC (A). The heatmap demonstrates the correlation between immune checkpoint genes and hub genes in the Epi_C1_SPARC cluster (B). The scatter plot illustrates the correlation between HMGA2 and immune checkpoint genes (C).

**Supplementary Figure 4 Basic Information on T Cells and TCR.**

Feature plots of canonical marker genes were displayed (A). An overlap analysis of TCR across different HSCC lesion stages was shown (B). A bar graph illustrated the proportion of cell types within each epithelial cell cluster (C). The dynamics of clonotypes between three samples from Patient 2 were presented (D). The red font in the legend on the right indicates statistically significant differences in the distribution of this cell subpopulation across different disease stages. Changes in cellular distribution of TCR at different lesion stages (E).

**Supplementary Figure 5 B Cells enrichment results and BCR Basic Information.**

GO-BP and Reactome enrichment results for B cell subclusters were presented (A-B). Overlap analysis of BCR in different HSCC lesion stages was depicted (C). Length distribution of CDR3 sequences of IGH and IGL chains in different HSCC lesion stages was displayed (D). Changes in cellular distribution of BCR at different lesion stages (E).

**Supplementary Figure 6 Basic information of fibroblasts and selection of CAF_C2_MME clusters.**

Box plots suggested cell occupancy in the fibroblast cluster in different HSCC lesion stages (A). A Heatmap displayed the top 10 differentially expressed genes (DEGs) in fibroblast clusters (B). Kaplan-Meier survival curves were shown for HNSCC from the Cancer Genome Atlas. Intratumoral heterogeneity was estimated based on detected signals for alternative subtypes and was divided into two groups with high and low CAF_C2_MME (C). The Venn diagram showed the common genes in five enriched pathways within the CAF_C2_MME subcluster (D). The bar plot illustrated the expression of MMP genes in 33 human cancers obtained from the Cancer Genome Atlas (E).
