# Supplementary material for: Revealing molecular and cellular heterogeneity in hypopharyngeal carcinogenesis through single-cell RNA and TCR/BCR sequencing
Source: Front Immunol. 2024 Apr 24;15:1310376. doi: 10.3389/fimmu.2024.1310376 (PMC11076829; doi:10.3389/fimmu.2024.1310376)
Supplement: Supplementary file 2 [file Table_1.doc]

**Supplementary Table 1** Basic information on scRNA-seq and TCR/BCR

| **Sample**  **ID** | **Sample type** | **10X version*** | **Cells** | **Median UMI counts per cell** | **Median genes per cell** | **Estimated number of T cells** | **Median TRA UMIs per Cell** | **Median TRB UMIs per Cell** | **Estimated number of B cells** | **Median IGL UMIs per Cell** |
| --- | --- | --- | --- | --- | --- | --- | --- | --- | --- | --- |
| Cancer_1 | Tumour | V3 | 7926 | 4268 | 1864 | 3006 | 8 | 17 | 889 | 318 |
| Cancer_2 | Tumour | V3 | 7272 | 7837 | 2819 | 535 | 5 | 15 | 68 | 2287 |
| Cancer_3 | Tumour | V3 | 8080 | 3862 | 1636 | 2024 | 5 | 14 | 333 | 27 |
| HGIN_1 | HGIN | V3 | 9734 | 3556 | 1681 | 2350 | 5 | 17 | 622 | 1314 |
| HGIN_2 | HGIN | V3 | 7634 | 3033 | 1342 | 1524 | 4 | 14 | 364 | 632 |
| HGIN_3 | HGIN | V3 | 8078 | 3782 | 1516 | 1846 | 3 | 10 | 1219 | 2418 |
| LGIN_1 | LGIN | V3 | 9071 | 4484 | 1813 | 4136 | 4 | 13 | 1107 | 94 |
| Normal_1 | Normal | V3 | 8183 | 3967 | 1703 | 2941 | 4 | 12 | 279 | 10612 |
| Normal_2 | Normal | V3 | 6882 | 3641 | 1477 | 1003 | 3 | 9 | 401 | 4162 |
| *Single-cell suspensions were converted into single-cell RNA-seq libraries using a commercially available DROP-seq protocol (10X genomics) following version 3 chemistry. HGIN, High-grade intraepithelial neoplasia; LGIN, Low-grade intraepithelial neoplasia; UMI, unique molecular identifier, equivalent to a unique detected transcript. | | | | | | | | | | |
