# Supplementary material for: Revealing molecular and cellular heterogeneity in hypopharyngeal carcinogenesis through single-cell RNA and TCR/BCR sequencing
Source: Front Immunol. 2024 Apr 24;15:1310376. doi: 10.3389/fimmu.2024.1310376 (PMC11076829; doi:10.3389/fimmu.2024.1310376)
Supplement: Supplementary file 3 [file Table_2.doc]

**Supplementary Table 2** CDR3 shared information of T cells in HSCC

| **CDR3 a chain** | **VDJ database** | **CDR3 b chain** | **VDJ database** |
| --- | --- | --- | --- |
| CAASDGTGKLIF | No | CASSEWAGGGYTF | No |
| CAATGSNSGGSNYKLTF | No | CASSLSSGRFDTQYF | No |
| CAFMRRDSNYQLIW | No | CASVRGTGELFF | No |
| CAGNNARLMF | EBV | CASSYGEEETQYF | No |
| CALSEASSGNTPLVF | CMV | CASSTFGGTDTGELFF | No |
| CAMGNSGYALNF | No | CASSPGGGNQPQHF | CMV |
| CAMTTDSWGKFQF | No | CASSLTRDEEKLFF | No |
| CASYGGSQGNLIF | CMV | CATSVTSYNEQFF | No |
| CATALSLNNNARLMF | No | CASSLETGRHYGYTF | No |
| EBV:Epstein-Barr virus，CMV:Cytomegalovirus | | | |
