# Supplementary material for: Revealing molecular and cellular heterogeneity in hypopharyngeal carcinogenesis through single-cell RNA and TCR/BCR sequencing
Source: Front Immunol. 2024 Apr 24;15:1310376. doi: 10.3389/fimmu.2024.1310376 (PMC11076829; doi:10.3389/fimmu.2024.1310376)
Supplement: Supplementary file 4 [file Table_3.doc]

**Supplementary Table 3** Clinical characteristics of 5 HSCC patients used for experimental verification in this study

| **Patients ID** | **Age** | **Gender** | **Sampling locations** |
| --- | --- | --- | --- |
| P1 | 71 | male | Posterior wall of the hypopharynx |
| P2 | 52 | male | Left pyriform fossa |
| P3 | 57 | male | Left pyriform fossa |
| P4 | 60 | male | Left pyriform fossa |
| P5 | 65 | male | Left pyriform fossa |
