# Supplementary material for: Revealing molecular and cellular heterogeneity in hypopharyngeal carcinogenesis through single-cell RNA and TCR/BCR sequencing
Source: Front Immunol. 2024 Apr 24;15:1310376. doi: 10.3389/fimmu.2024.1310376 (PMC11076829; doi:10.3389/fimmu.2024.1310376)
Supplement: Supplementary file 5 [file Table_4.doc]

**Supplementary Table 4** Primers Used for qRT-PCR Analysis of Target Genes

| Gene | Forward Primer | Reverse Primer |
| --- | --- | --- |
| hMAGEA3 | 5’-GTTCCAAGCAGCACTCAGTA-3’ | 5’-TCCACTTCCATCAGCTCG-3’ |
| hMMP7 | 5’-TACTCGAGACTTACCGCAT-3’ | 5’-GCAAAGCCAATCATGATGT-3’ |
| hMMP3 | 5’-ACTCACATTCTCCAGGCTGT-3’ | 5’-AGTGGCCAATTTCATGAGC-3’ |
| GAPDH | 5’- GCCTTCCGTGTCCCCACTGC-3’ | 5’- GGCTGGTGGTCCAGGGGTCT-3’ |
