# Supplementary figures and images for: Revealing molecular and cellular heterogeneity in hypopharyngeal carcinogenesis through single-cell RNA and TCR/BCR sequencing
Source: Front Immunol. 2024 Apr 24;15:1310376. doi: 10.3389/fimmu.2024.1310376 (PMC11076829; doi:10.3389/fimmu.2024.1310376)

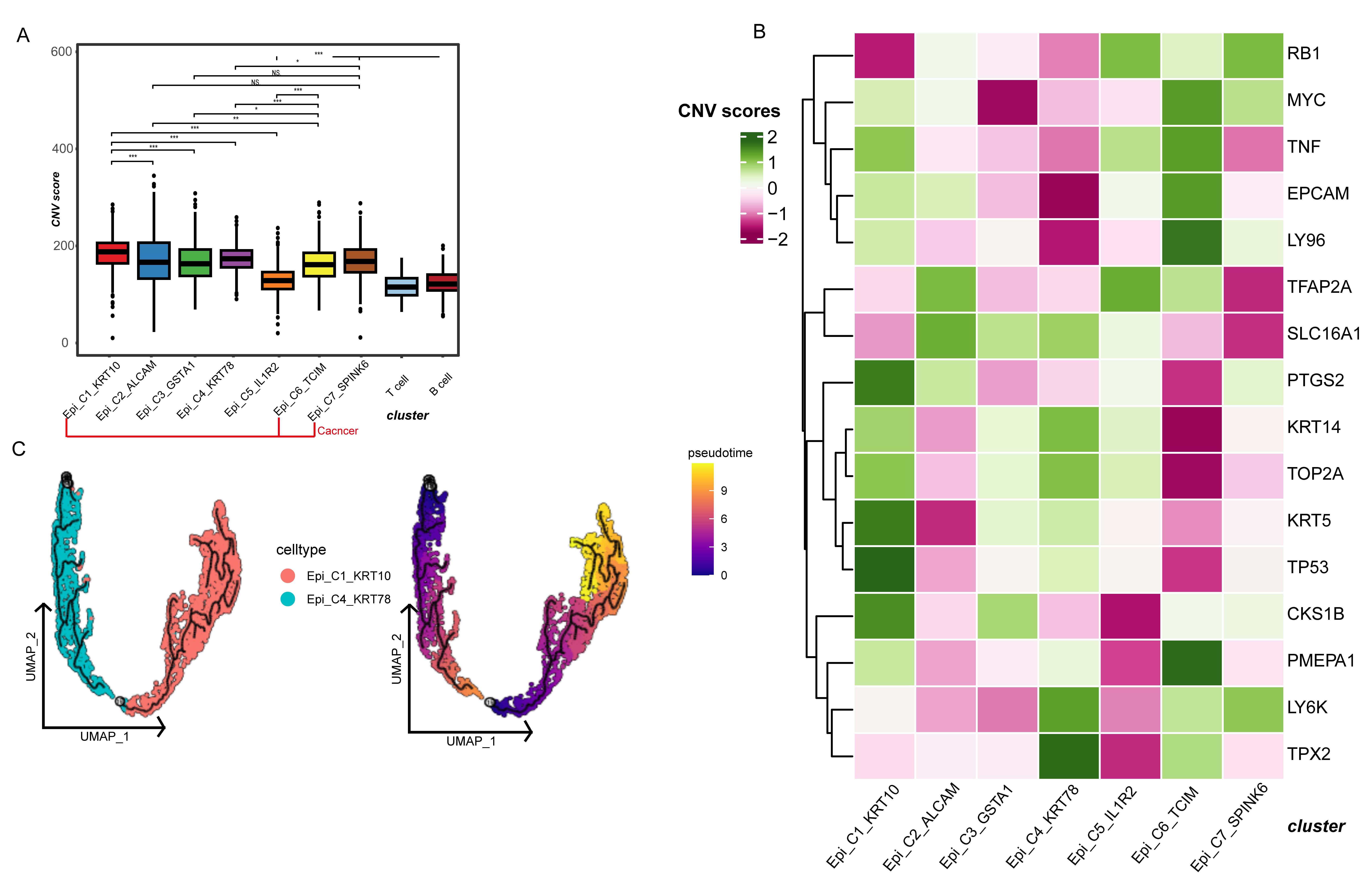

Supplement: Supplementary file 8 [file Image_2.tif]

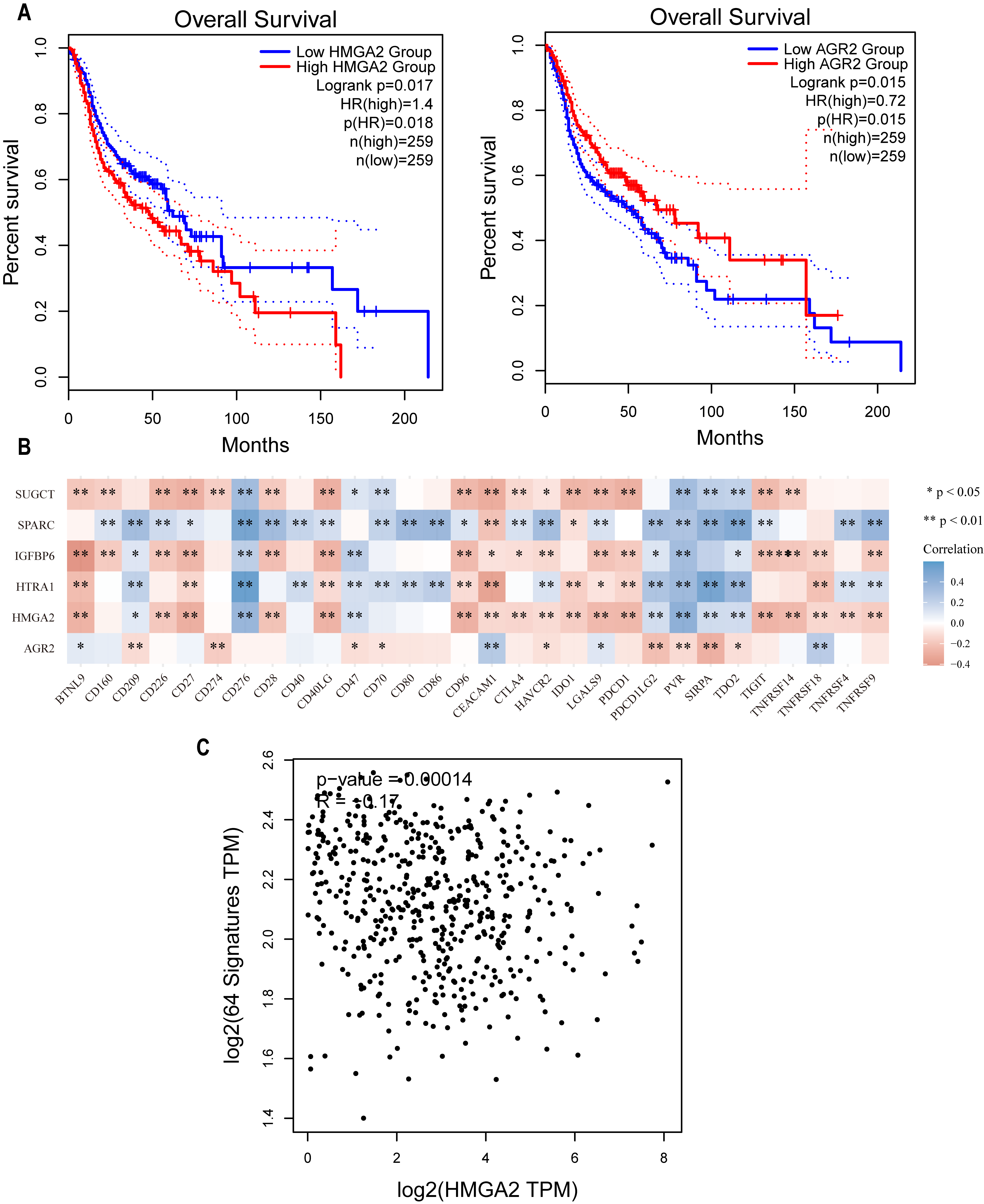

Supplement: Supplementary file 9 [file Image_3.tif]

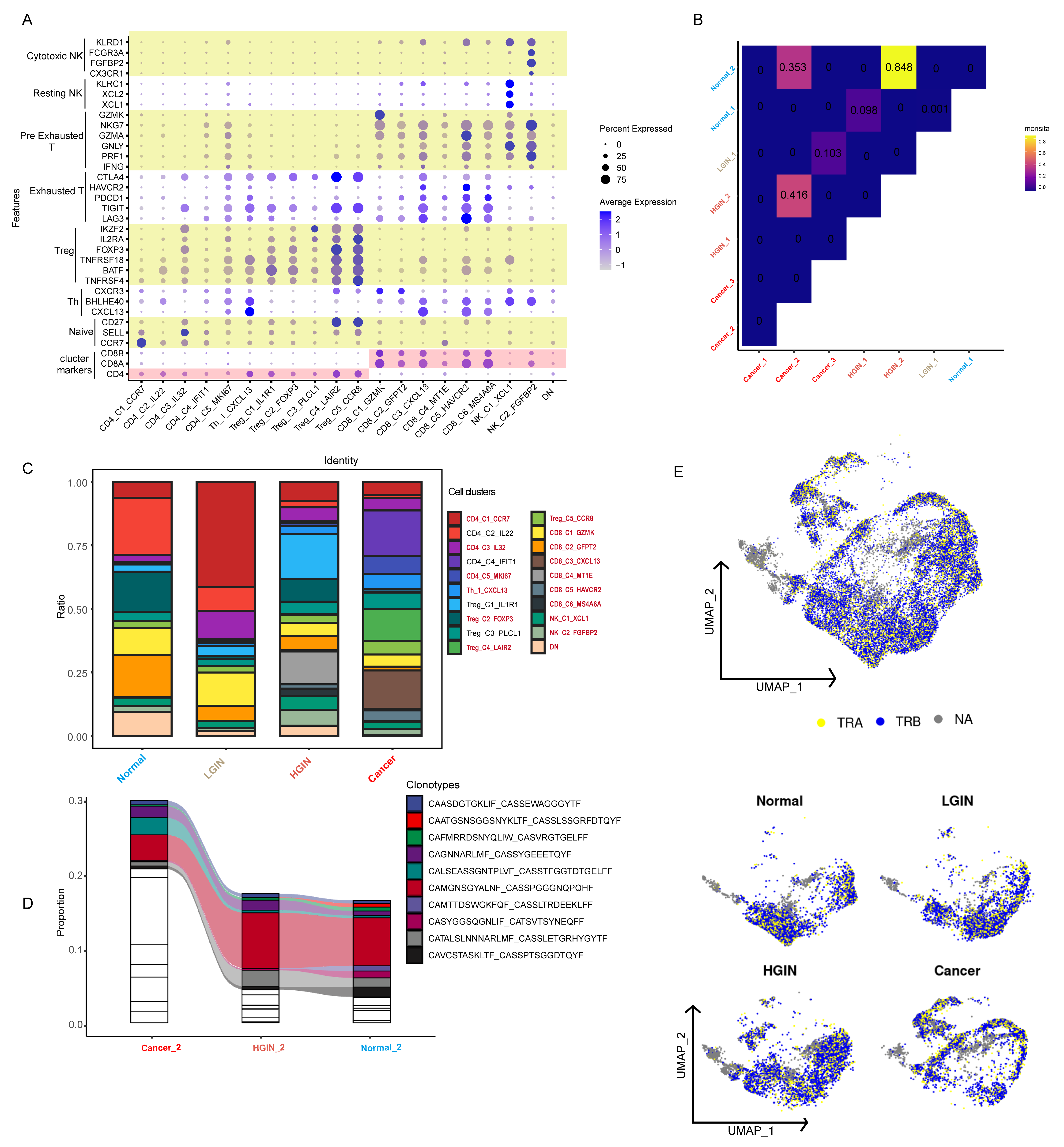

Supplement: Supplementary file 10 [file Image_4.tif]

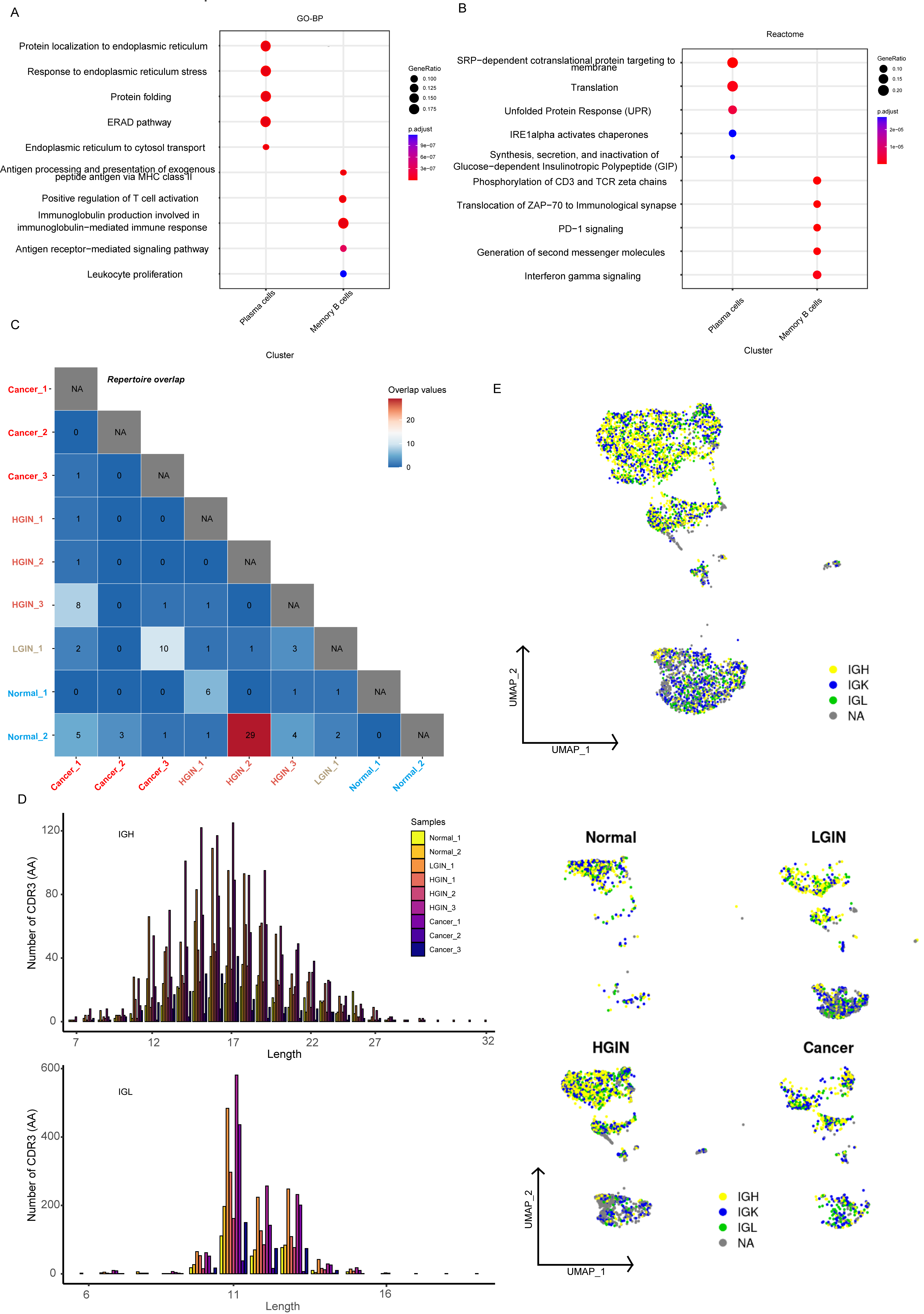

Supplement: Supplementary file 11 [file Image_5.tif]

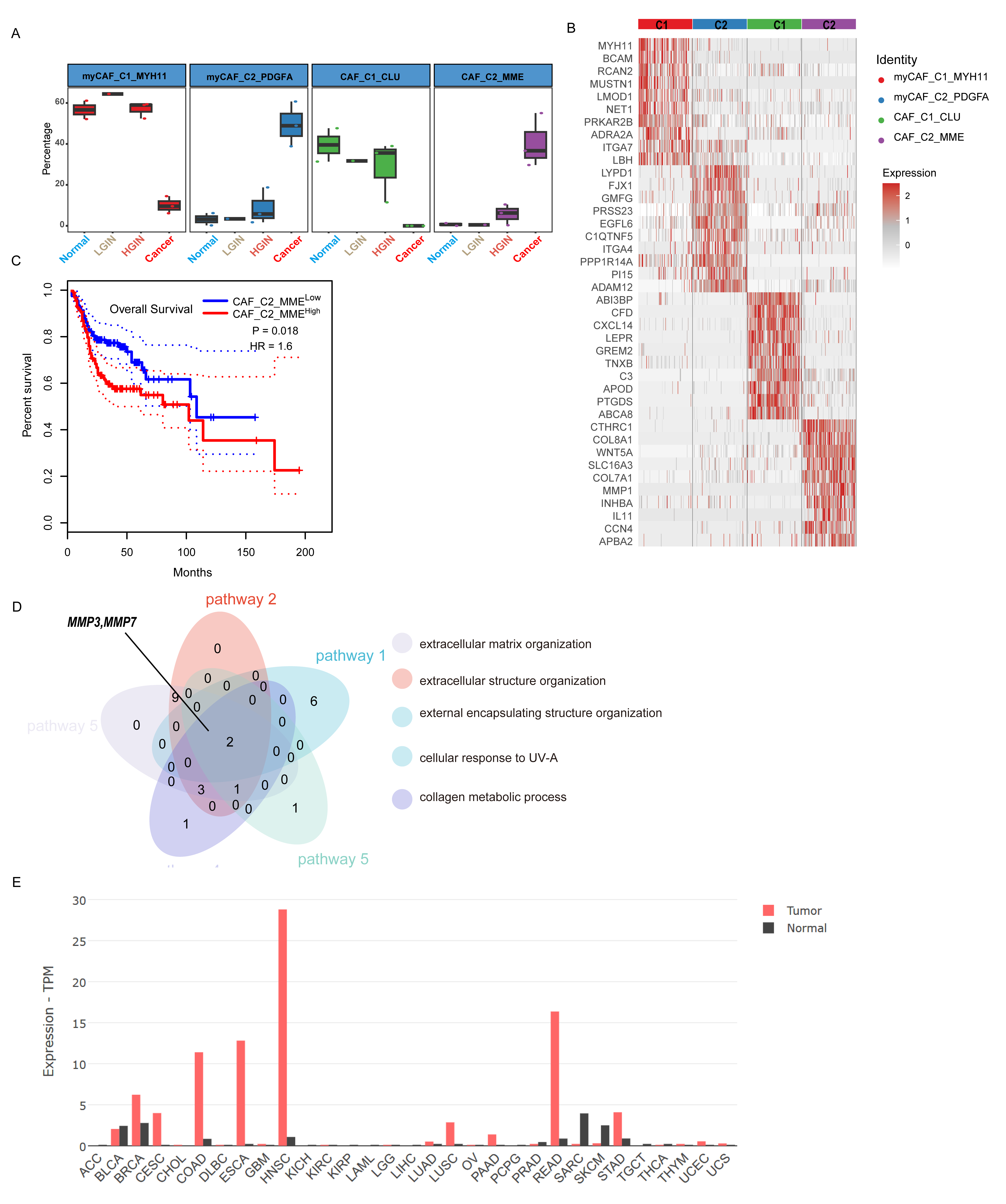

Supplement: Supplementary file 12 [file Image_6.tif]
